# Neurofibromas of the bladder in a child with neurofibromatosis type 1

**DOI:** 10.1590/S1677-5538.IBJU.2017.0199

**Published:** 2018

**Authors:** Gulec Mert Dogan, Ahmet Siğirci, Leyla Karaca

**Affiliations:** 1 Department of Radiology Pediatric, Inonu University Malatya, Turkey

## CASE DESCRIPTION

**Figure 1:**
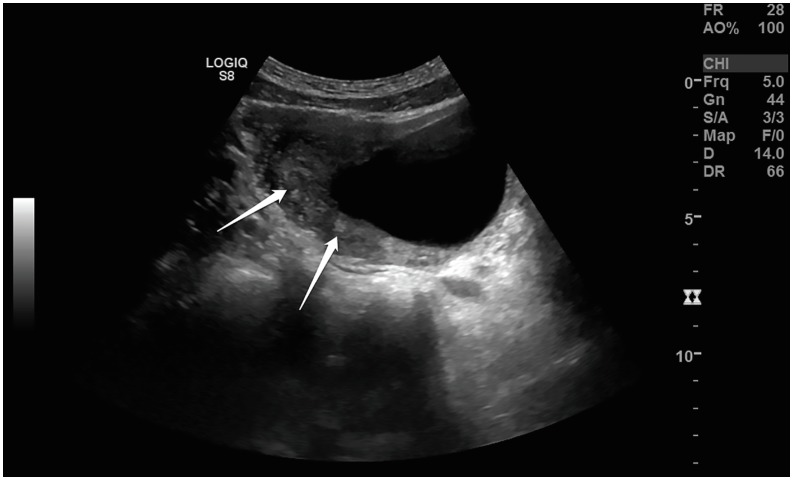
Ultrasound imaging; Diffuse thickening of the anterosuperior and posterior walls of the bladder and multiple round, <5mm nodular echogenities in the thickened walls marked with arrows.

**Figure 2:**
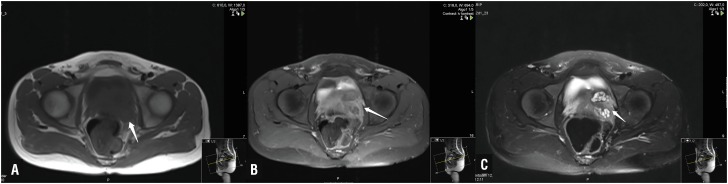
MRI axial images; nodular lesions marked with arrows. A. T1 weighted B. post contrast T1 weighted fat suppressed C. T2 weighted fat suppressed image.

**Figure 3:**
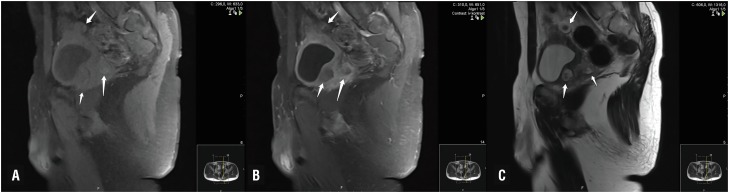
MRI sagittal images; nodular lesions marked with arrows A. T1 weighted fat suppressed B. Post contrast T1 weighted fat suppressed C. T2 weighted image.

A 17-year-old boy diagnosed with neurofibromatosis type 1 (NF1) presented with a six-month history of hematuria, dysuria, and urinary frequency. Ultrasonography (USG) revealed diffuse thickening of the anterosuperior and posterior walls of the bladder with round, <5mm nodular echogenities in the thickened walls (Figure-1). Magnetic resonance imaging (MRI) of the pelvis revealed a nodular lesion with low signal intensity on T1 and fat suppressed T1 weighted (T1-W) images; and nodular lesions with a ‘target sign’ on T2 weighted (T2-W) images. This consisted of low signal intensity fibrosis surrounded by high signal intensity stroma at the posterior of the bladder wall (Figures 2 and 3). The patient's symptoms were relieved after antibiotic treatment and he has had no serious complaints since then. He is now monitored by the urology outpatient clinic.

Children with NF1 should always be evaluated for neurofibromatosis of the genitourinary system (1). Bladder involvement of neurofibromatosis is rare and presenting features include irritative voiding symptoms and hematuria due to recurrent urinary tract infections (2). On USG, bladder involvement of neurofibromas can manifest as a focal mass or as diffuse bladder wall thickening. On MRI, neurofibromas display low-signal intensity on T1-W images and non-homogeneous high-signal intensity with a ‘target sign’ on T2-W images (3). Differential diagnosis includes rhabdomyosarcoma, ganglioneuroma, and retroperitoneal fibrosis (4). In a patient with NF1, the primary consideration should be neurofibroma. Generally, management of patients with NF1 and bladder involvement is conservative. If there are intractable symptoms such as hydronephrosis, bladder volume loss and suspicion for malignant degeneration, surgical treatment may be needed (3).

In conclusion, conventional MRI and ultrasound are important imaging modalities for the evaluation of genitourinary involvement of neurofibromatosis disease type 1.
